# Follicular lymphoma presenting as scalp mass deformity: Case Report and Review of the literature

**DOI:** 10.15761/BRCP.1000155

**Published:** 2018-02-24

**Authors:** Divine Nwafor, Walid Radwan, Brandon Lucke-Wold, William Underwood, Kymberly Gyure, Robert Marsh

**Affiliations:** 1 Department of Neurosurgery, School of Medicine, West Virginia University, Morgantown, WV, USA; 2 Department of Neurosurgery, University of Michigan, Ann Arbor, MI, USA; 3 Department of Pathology, School of Medicine, West Virginia University, Morgantown, WV, USA

**Keywords:** scalp mass, follicular lymphoma, radiotherapy, chemo-immunotherapy

## Abstract

Lymphoma presenting as a scalp mass is a rare but serious medical condition mandating aggressive treatment and neurosurgical intervention. We report a case of 53-year-old male who presented with a large right sided frontal scalp mass and a smaller mass located on the left frontal scalp. After discussion with the patient, it was decided to resect the larger mass for definitive diagnosis. After subtotal resection of the mass, biopsy revealed WHO grade 1 follicular lymphoma (FL), diffuse pattern stage IV. The patient was subsequently treated with 4 grays (Gy) of palliative radiotherapy over 2 fractions to the right frontal scalp and systemic chemo-immunotherapy (6 cycles) followed by rituximab maintenance. Lumbar puncture to obtain cerebrospinal fluid was done a month after therapy began and the results were negative for spread of malignant cells. Approximately 3 months after initiation of therapy, PET/CT showed no evidence of active malignancy and MRI revealed a complete internal resolution of the enlarged right frontal scalp mass. We use this case to provide a detailed discussion regarding disease pathophysiology, early diagnosis, and management

## Introduction

Non-Hodgkin’s lymphoma (NHL) is a broad class of malignant neoplasms originating from B-cell progenitors, T-cell progenitors, mature B-cells, mature T-cells, and in rare cases natural killer cells (NK-cells) [1]. The second most common type of NHL is follicular lymphoma (FL). FL has been described as a heterogeneous malignancy that includes tumors often derived from germinal center B-cells, centrocytes, and occasionally centroblasts [2]. There are 3.18 cases of FL per 100,000 people seen in the United States with a greater predilection (2X) in Caucasian than African-American or Asian populations [3]. Patients diagnosed with this malignancy are often asymptomatic and do not present with the B symptoms of fever, weight loss, and night sweats. Because of its indolent nature, the disease (FL) may have already disseminated to other regions at the time of diagnosis [4]. We discuss a patient with a rare disseminated scalp follicular lymphoma and look at the histopathology associated with this patient’s disease. Furthermore, we use the case to illustrate the importance of early detection and appropriate clinical management.

## Case report

**Figure 1:**
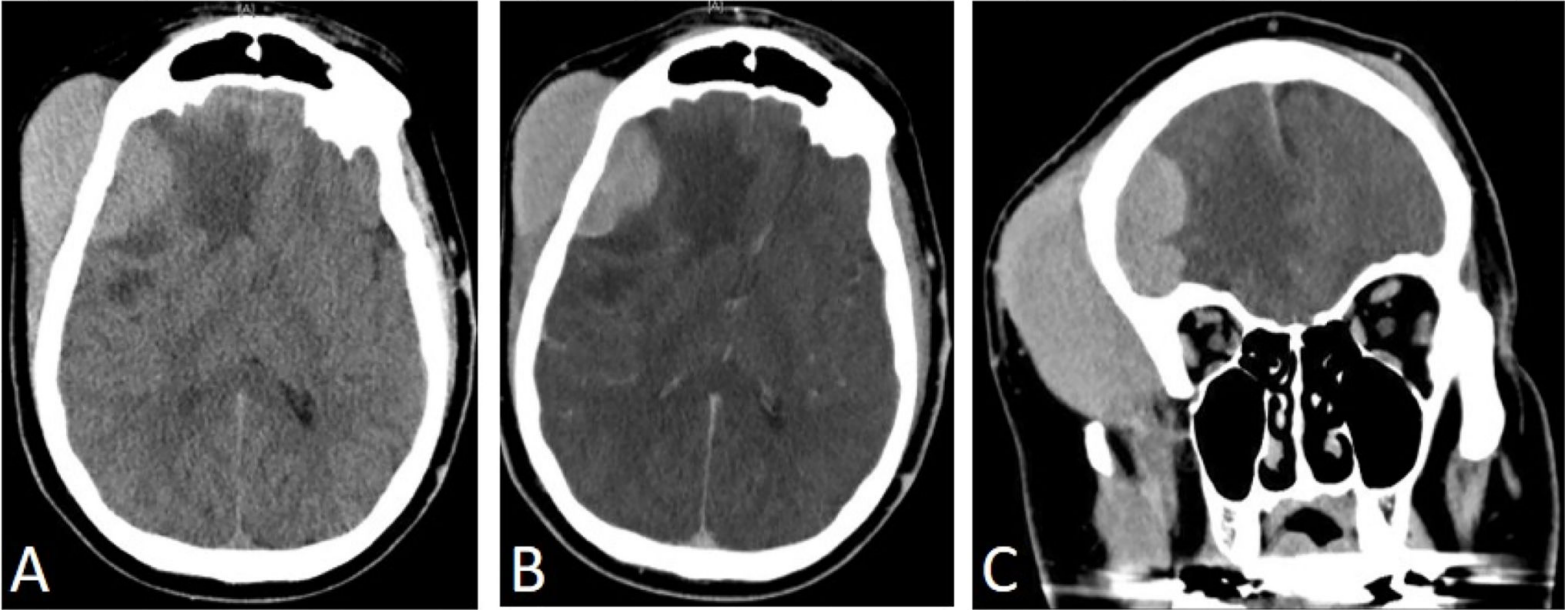
CT head showing enlarged right frontal scalp mass. A: axial view non-contrast, B: axial view with contrast, C: Coronal view with contrast.

**Figure 2:**
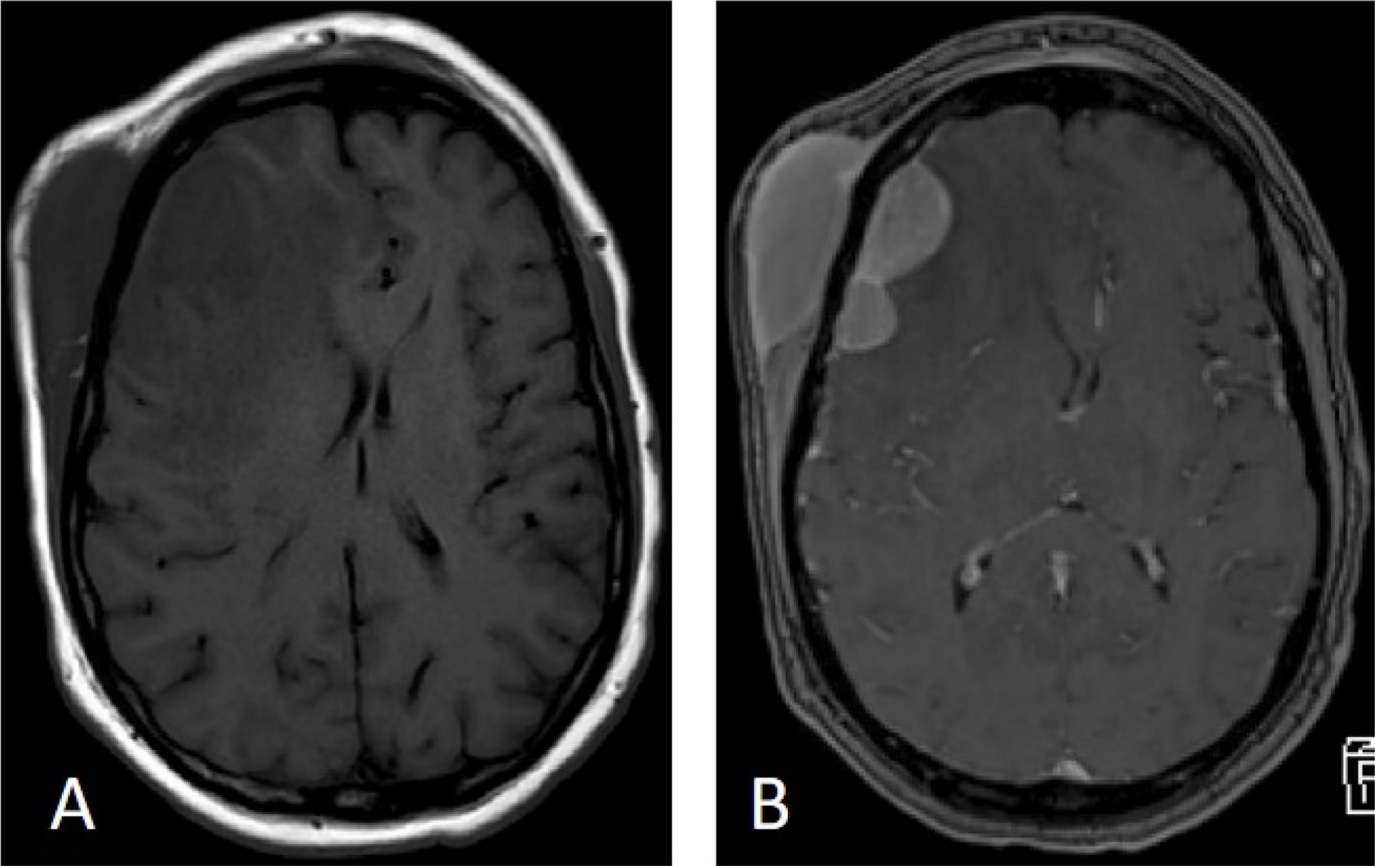
MRI showing scalp mass, vasogenic edema, and midline shift. A: Axial view T1 no contrast, B: Axial view T1 with contrast.

**Figure 3:**
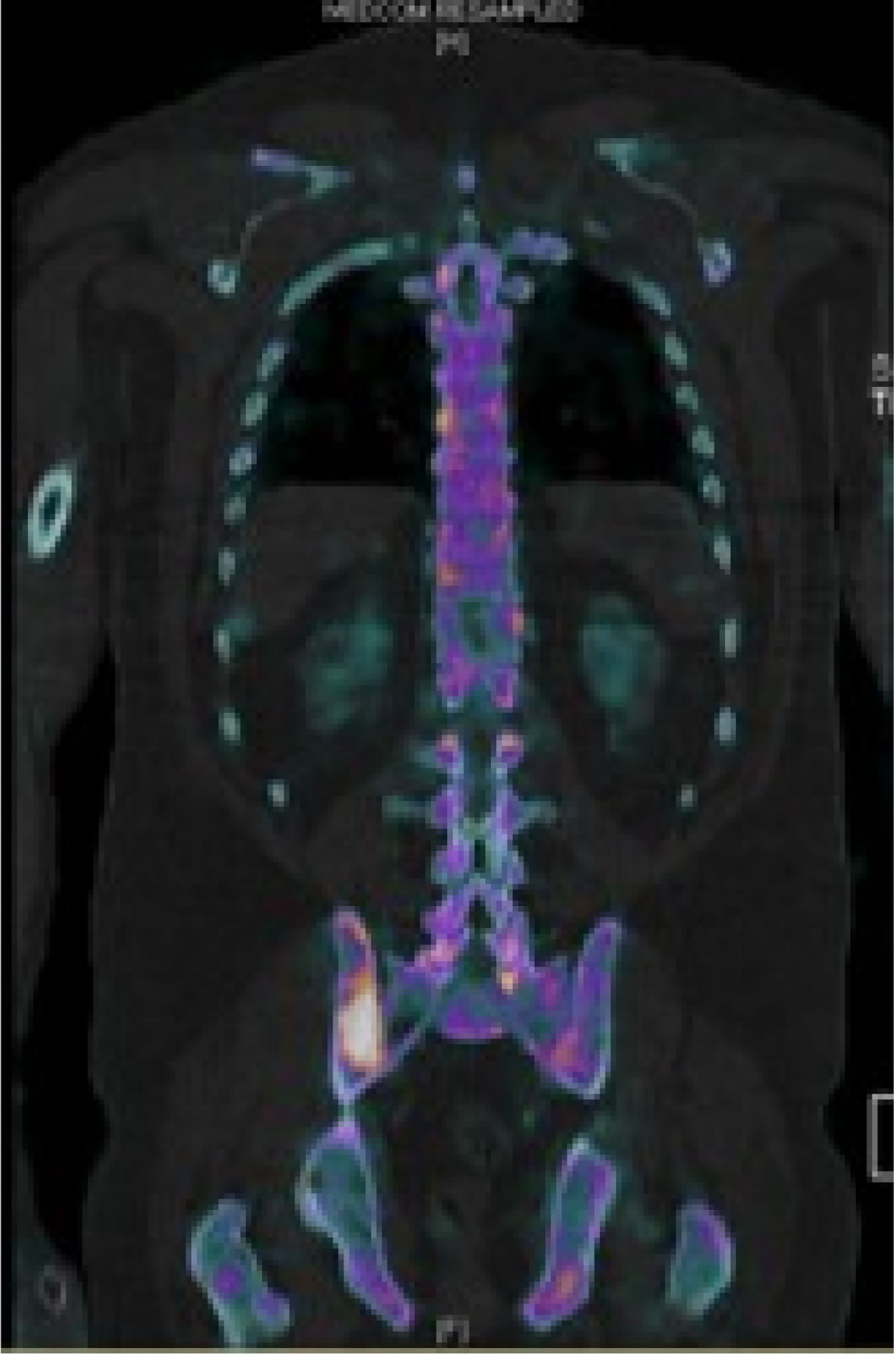
Nuclear medicine bone scan with 99mTc-hydroxymethylene diphosphonate (HMDP) and SPECT showed multiple areas of uptake concerning for malignancy. Uptake shown in right posterior iliac bone.

Our patient is a 53-year-old male who worked as a coal miner. In spring of 2017, he noticed enlarging scalp nodules and right hip pain. He also complained of fatigue but was otherwise asymptomatic. Because of the hip pain and nodules, he came to the emergency department where a CT scan showed an enlarged right frontal scalp mass that measured about 4 cm (Figure 1). MRI also revealed a right frontal scalp mass and extensive vasogenic edema localized to the right frontal lobe with a 5 mm midline shift at the foramen of Monroe (Figure 2). A subsequent CT chest, abdomen, and pelvis was performed and showed a lytic lesion in his ilium and a single enlarged right hilar lymph node (not shown). A follow-up nuclear medicine bone scan demonstrated multiple areas of increased uptake concerning for metastasis (Figure 3). One of the largest areas was in the right frontal scalp. After discussion with the patient and the need for a definitive diagnosis, he was consented for subtotal resection of the right frontal scalp mass.

**Figure 4:**
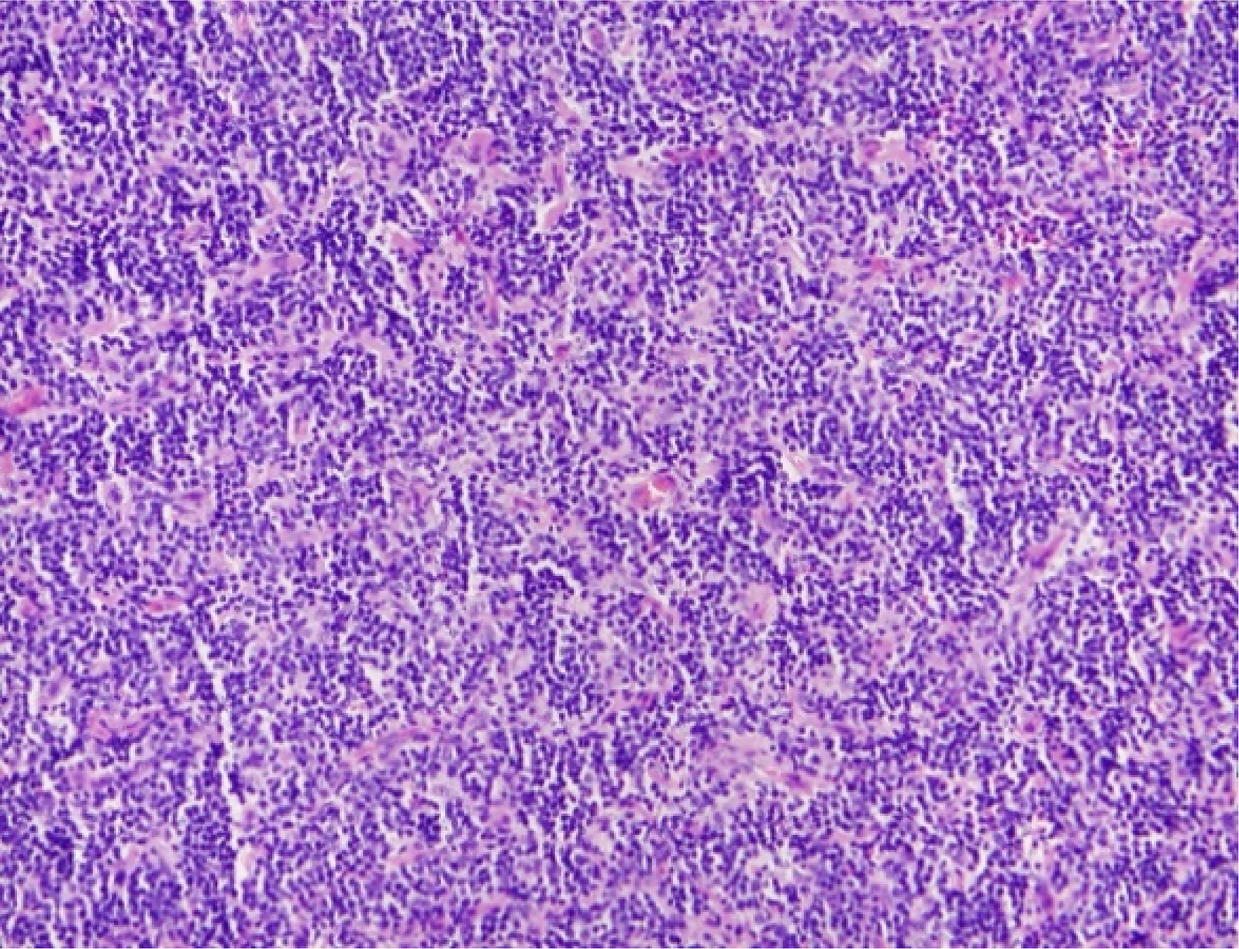
Histopathologic staining showing monotonous infiltrate composed of small cells with irregular nuclei, condensed chromatin, and inconspicuous nucleoli.

**Figure 5:**
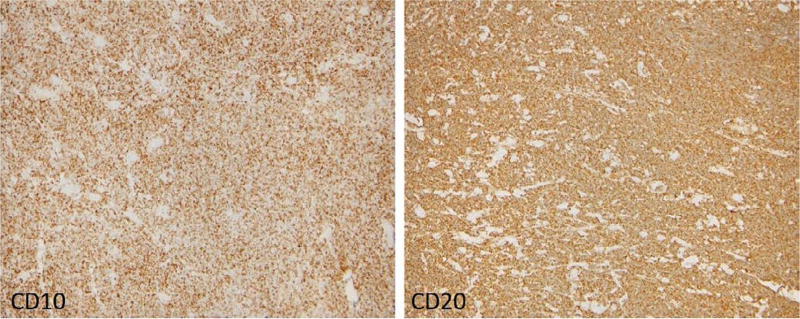
Immunohistochemistry showing cells that are CD20 positive and co-express CD10 and Bcl2.

He was taken to the operating room where a right frontal scalp incision was performed for subtotal resection of likely metastatic cancer. The mass was primarily located within the right frontal scalp but extended through the skull into the right frontal cortex. It did not however appear to be involved with the brain parenchyma. The resection consisted of removing scalp mass and taking a core biopsy from the specimen. Histopathology of the biopsy showed a diffuse and monotonous infiltrate composed of small cells with irregular nuclei, condensed chromatin and inconspicuous nucleoli (Figure 4). On immunohistochemistry, the cells are CD20 positive B-cells and co-express CD10 and Bcl-2 but do not express CD3, 5, 23, 43 and cyclin D1 (Figure 5). CD21 was present highlighting a disrupted follicular dendritic cell meshwork. Ki-67 proliferation rate was low (less than 20%). By flow cytometry, a dim kappa light chain-restricted B-cell population was identified expressing CD10. The FISH assay revealed t(14;18) *IGH* (immunoglobulin heavy chain locus)-Bcl-2 fusion. These findings support the definitive diagnosis of follicular lymphoma, WHO grade 1, diffuse pattern stage IV.

After definitive diagnosis was made, the patient was then referred to radiation oncology for follow-up. By time of presentation to radiation oncology, the patient was showing symptoms of lower extremity numbness, personality changes, and some memory loss. The radiation oncologist discussed the diagnosis with the patient and his family and came up with a treatment plan to combat the unusual presentation of follicular lymphoma with the intracranial extension from the calvarium. The treatment plan consisted of repeat MRI’s; local (palliative) XRT (4 Grays over two fractions) and systemic chemo-immunotherapy (6 cycles) followed by rituximab maintenance. Lumbar puncture was done a month after resolution of vasogenic edema to assess leptomeningeal spread later proven negative for malignant cells. MRI showed complete resolution of the right scalp mass post-radiotherapy and after the patient had already underwent 3 cycles of chemo-immunotherapy. PET/CT done approximately 3 months since therapy initiation showed no suspicion for abnormal hypermetabolic activity, which suggested the absence of active malignancy.

## Discussion

In the United States, FL represents 35% of all NHL diagnoses with the majority being sporadic [3]. Occasionally some types of FL have been associated with rare genetic mutations [5]. For the sporadic subtype there have been numerous risk factors proposed such as drugs, toxins, diseases, and infectious agents but how much they actually contribute to the development of FL is unknown due to the lack of prospective studies [6]. Other factors that have a stronger association include; age (FL is rare in children) and ethnicity (more common in Caucasians) [7]. The disease is often initially suspected by symptoms but can remain undiagnosed for months to years. The most common symptom for FL patients is painless adenopathy in the hilar and mediastinal nodes. Unfortunately, only 20% of FL patients have B-symptoms such as fatigue and night sweats [1]. The lack of symptoms can contribute to the widespread metastatic distribution commonly seen at initial presentation. Once suspected by the clinician, definitive diagnosis is best achieved by biopsy of a suspected lesion or lymph node.

Our patient was unique in that he presented with a rare type of FL that had spread to the scalp by time of symptom onset. An extensive case series of FL patients showed that of 93 cases only one patient had metastatic spread to the neck with none to the scalp highlighting the rarity of our case [8]. The reason for the rapid spread in our patient was likely due to the type of FL with diffuse pattern staining on histopathology. The higher number of centroblasts makes the cancer more aggressive compared to FLs with centrocyte predominance [9]. Furthermore, our patient had a t(14:18) translocation. As a consequence of this translocation, Bcl-2 comes under the control of the *IGH* enhancer, causing constitutive expression of the antiapoptotic protein BCL2 [10]. Dissemination to organs that do not have bone marrow or lymphatic predominance is highly uncommon. The standard for treatment once metastasis does occur is resection, radiation, chemotherapy, and then rituximab [9]. Despite being a scalp mass, our patient received this same regimen. Some groups are currently looking at interferon alpha 2a therapy for treatment, but exactly how this therapy works is still being investigated [11]. The key for management is early detection and aggressive resection followed by radiation and chemotherapy. Long-term prognosis with scalp metastasis is still unknown due to rarity of the disease phenotype [4].

In summary, our patient had an aggressive metastatic FL that spread to his scalp. While initially asymptomatic he quickly progressed to B symptoms. Surgical resection, radiation, chemotherapy, and rituximab were used successfully to manage the FL. 3 months since therapy the patient is doing well. The key points going forward are related to the need for future study. First, understanding FL pathophysiology is critical for developing disease classification criteria. Second, prospective studies are needed to map survival once scalp metastasis has occurred. Finally, experimental immune modulator therapies should be explored further as adjuvant treatment options. With these three approaches improved management for patients with aggressively expanding FLs can be obtained.

## References

[1] Shankland KR, Armitage JO, Hancock BW (2012). Non-Hodgkin lymphoma. Lancet.

[2] El Behery R, Laurini JA, Weisenburger DD, Smith LM, Dave BJ (2018). Follicular large cleaved cell (centrocytic) lymphoma: an unrecognized variant of follicular lymphoma. Hum Pathol.

[3] Morton LM, Wang SS, Devesa SS, Hartge P, Weisenburger DD (2006). Lymphoma incidence patterns by WHO subtype in the United States, 1992–2001. Blood.

[4] Anderson T, DeVita VT, Simon RM, Berard CW, Canellos GP (1982). Malignant lymphoma. II Prognostic factors and response to treatment of 473 patients at the National Cancer Institute. Cancer.

[5] Ebrahim AS, Kandouz M, Emara N, Sugalski AB, Lipovich L (2017). Unintended target effect of anti-BCL-2 DNAi. Cancer Manag Res.

[6] Negri E, Little D, Boiocchi M, La Vecchia C, Franceschi S (2004). B-cell non-Hodgkin’s lymphoma and hepatitis C virus infection: a systematic review. Int J Cancer.

[7] Pinto A, Hutchison RE, Grant LH, Trevenen CL, Berard CW (1990). Follicular lymphomas in pediatric patients. Mod Pathol.

[8] Khatib Y, Dande M, Patel RD, Makhija M (2017). Primary cutaneous large B-cell lymphoma of scalp: Case report of a rare variant. Indian J Pathol Microbiol.

[9] Swerdlow SH, Campo E, Pileri SA, Harris NL, Stein H (2016). The 2016 revision of the World Health Organization classification of lymphoid neoplasms. Blood.

[10] Rowley JD (1988). Chromosome studies in the non-Hodgkin’s lymphomas: the role of the 14;18 translocation. J Clin Oncol.

[11] Kimby E, Jurlander J, Geisler C, Hagberg H, Holte H (2008). Long-term molecular remissions in patients with indolent lymphoma treated with rituximab as a single agent or in combination with interferon alpha-2a: a randomized phase II study from the Nordic Lymphoma Group. Leuk Lymphoma.

